# The effect of 12 weeks of combined upper- and lower-body high-intensity interval training on muscular and cardiorespiratory fitness in older adults

**DOI:** 10.1007/s40520-018-1015-9

**Published:** 2018-07-26

**Authors:** Christopher Hurst, Kathryn L. Weston, Matthew Weston

**Affiliations:** 1 School of Applied Social Sciences, Durham University, Durham, UK; 2 Department of Psychology, Sport and Exercise, School of Social Sciences, Humanities and Law, Teesside University, Middlesbrough, UK; 3 School of Health and Social Care, Teesside University, Middlesbrough, UK

**Keywords:** High-intensity interval training, Muscular strength, Muscular power, Physical performance, Cardiorespiratory fitness, Ageing

## Abstract

**Background:**

High-intensity interval training (HIT) can impact cardiorespiratory and muscular fitness simultaneously, yet protocols typically focus on lower-body exercise. For older adults however, performing activities of daily living requires upper- and lower-body fitness.

**Aims:**

To assess the effects of combined upper- and lower-body HIT on fitness in adults aged > 50 years.

**Methods:**

Thirty-six adults (50–81 years; 21 male) were assigned via minimisation to either HIT (*n* = 18) or a no-exercise control group (CON, *n* = 18) following baseline assessment of leg extensor muscle power, handgrip strength, cardiorespiratory fitness (predicted *V*O_2max_) and health-related quality of life (HRQoL). The HIT group completed two training sessions per week for 12-weeks, performing a combination of upper-, lower- and full-body exercises using a novel hydraulic resistance ergometer. Data were analysed via ANCOVA with probabilistic inferences made about the clinical relevance of observed effects.

**Results:**

All participants completed the intervention with mean (82 ± 6%HR_max_) and peak (89 ± 6%HR_max_) exercise heart rates confirming a high-intensity training stimulus. Compared with CON, HIT showed possibly small beneficial effects for dominant leg power (10.5%; 90% confidence interval 2.4–19.4%), non-dominant leg power (9.4%; 3.3–16.0%) and non-dominant handgrip strength (6.3%; 1.2–11.5%) while the intervention effect was likely trivial (5.9%; 0.5–11.5%) for dominant handgrip strength. There was a likely small beneficial effect for predicted *V*O_2max_ (8.4%; 1.8–15.4%) and small-moderate improvements across several domains of HRQoL.

**Conclusion:**

Combined upper- and lower-body HIT has small clinically relevant beneficial effects on muscular and cardiorespiratory fitness in older adults.

**Electronic supplementary material:**

The online version of this article (10.1007/s40520-018-1015-9) contains supplementary material, which is available to authorized users.

## Introduction

Age-associated physiological changes in the neuromuscular and cardiorespiratory systems have important implications for maintaining an independent and healthy life into old age. Reduced muscular fitness is indicative of functional limitation [1] with lower-body muscle power output of particular relevance because of its association with the capacity to perform the activities of daily living [2–4]. Moreover, reduced cardiorespiratory fitness (*V*O_2max_) is associated with an increased risk of morbidity and mortality [5]. Exercise training offers a potential strategy for counteracting the deleterious effects of ageing with endurance and strength training capable of eliciting substantial fitness improvements in older adults [6, 7]. However, the divergent nature of physiological adaptation induced via endurance training and strength training means that older adults should perform both exercise modalities to maximise potential health and fitness benefits [8]. Despite this, the requirement to perform separate endurance and strength training activities places considerable time demands on individuals—an important consideration as lack of time remains a frequently cited barrier to exercise in a population where adherence to exercise guidelines remains poor [9, 10]. As such, training interventions which can elicit the benefits of endurance and strength training within a single exercise session may be an attractive proposition for potential exercisers.

High-intensity interval training (HIT), characterised by brief, intermittent bursts of vigorous activity interspersed with periods of rest or low-intensity exercise [11] offers an appealing training strategy because of its potential to induce positive adaptations in muscular and cardiorespiratory fitness simultaneously [12]. In older adults, HIT has previously been shown to be an effective strategy to improve cardiorespiratory [13, 14], muscular [15, 16] and functional fitness [17] with potentially greater effects for older and less fit individuals [18].

Despite emerging evidence supporting the effectiveness of HIT [15, 16], previous investigations have typically utilised cycle ergometry or treadmill walking/running as the preferred exercise mode, thereby providing a predominantly lower-body training stimulus. This is likely to be a sub-optimal training approach in this population as older adults need to maintain both upper- and lower-body fitness to perform the basic activities of daily living [1, 19], thereby suggesting a need for alterative exercise modes for performing HIT. Moreover, training modes which focus on lower-body exercise may be unsuitable or prohibitive for older adults with lower-body musculoskeletal or mobility complications. As such, innovative approaches to delivery of HIT are needed to increase accessibility for a greater number of individuals. Our study therefore, sought to evaluate the effects of 12-weeks of combined upper- and lower-body HIT performed using a novel exercise ergometer on measures of muscular and cardiorespiratory fitness as well as health-related quality of life in adults aged over 50 years.

## Methods

### Sample size estimation

Estimation of sample size for this investigation was performed using leg extensor muscle power as our primary outcome measure because of its importance for maintaining effective physical functioning in older adults [3, 4]. Sample size was estimated using a custom-made spreadsheet [20] by combining the smallest meaningful change in leg extensor power and the within-subject typical error as determined in our previous work [21]. Based on this, a sample size of 30 participants (15 per group) was recommended, however, we aimed to recruit 36 participants to allow for dropout during the intervention period.

### Participants

**Fig. 1:**
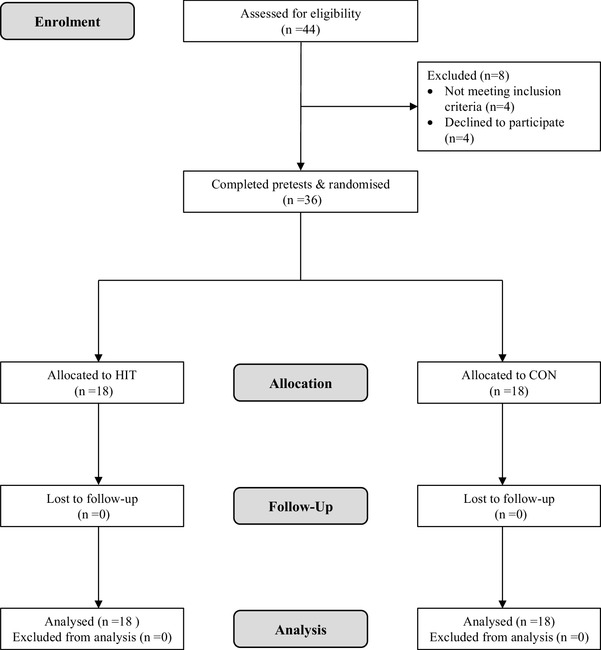
Flow of participants through the study

Initially, 44 potential participants were assessed for eligibility following recruitment via word of mouth and advertisement in local newspapers. Prior to enrolment, all potential participants completed a medical screening questionnaire to identify any medical conditions and current medication that could affect their ability to perform the required exercise training and testing. Those with pre-existing, neuromuscular or skeletal conditions, systemic disease (e.g., diabetes mellitus, cancer, heart disease) or those currently taking medications known to influence fitness or interpretation of the findings (*n* = 3) or who had engaged in formal and systematic (moderate to high-intensity) endurance or strength training within the last year were excluded (*n* = 1). Following explanation of the study protocol, four participants declined to take part in the investigation. Participants meeting the inclusion criteria (*n* = 36) were physically active but were not currently, and had not in the previous year, engaged in structured exercise more than twice per week. All individual participants provided written, informed consent to participate in the study (http://www.clinicaltrials.gov identifier NCT02714088) which conformed to the requirements of The Declaration of Helsinki and was approved by Teesside University Research and Ethics Committee. Figure 1 documents the flow of participants through the study.

### Experimental design

Following baseline testing (April 2016), participants were allocated to either HIT (*n* = 18) or no-exercise control (CON; *n* = 18) using the minimisation approach. This method aims to ensure balance between-groups at baseline across several prognostic factors even when sample sizes are small [22]. In this investigation, we defined these factors as age, sex, peak power of dominant leg, peak handgrip strength of dominant hand and aerobic fitness. Minimisation was performed using a custom-made spreadsheet [23] by an investigator not involved in baseline testing (MW) with individual subject data coded to ensure blinded allocation. Participant characteristics at baseline are presented in Table 1. All participants (HIT and CON) were instructed to maintain habitual physical activity and not engage in any additional structured exercise outside of the intervention.

**Table 1:** Participant characteristics at baseline

	HIT (*n* = 18)	CON (*n* = 18)
Age in years [mean (range)]	61.9 (50–81)	62.8 (50–74)
Sex (number male/female)	11/7	10/8
Height (cm)	167.6 ± 10.3	169.2 ± 9.4
Body mass (kg)	79.1 ± 14.4	78.9 ± 18.9
Body mass index (kg /m^2^)	28.1 ± 4.4	27.4 ± 5.3
Dominant leg extensor muscle power (W)	159.2 ± 64.8	161.8 ± 63.2
Dominant handgrip strength (kg)	36.2 ± 10.9	33.9 ± 11.0
Predicted *V*O_2max_ (mL · kg^−1^· min^−1^)	33.8 ± 8.3	33.9 ± 5.4

Data are presented as mean ± SD unless otherwise stated

*HIT* high-intensity interval training group, *CON* no-exercise control group

### HIT intervention

Participants allocated to HIT completed two instructor-led, combined upper- and lower-body HIT sessions per week for 12-weeks (April–August 2016), performed in groups of two to five participants with 72 h recovery between sessions. As the aim of this study was to evaluate the effectiveness of the exercise stimulus, effort was made to promote maximal attendance for all participants. As such, when a participant was unable to attend a session for reasons such as work, family commitments or illness, the session was rescheduled wherever possible. To be included in the final analysis, participants were required to attend a minimum of 90% (≥ 22/24) of sessions.

The HIT intervention was performed using a novel double-concentric, hydraulic resistance ergometer (Speedflex, AlphaTech Inc, Nelson, NC, USA). Each session began with a warm-up of progressive intensity (~ 6 min) consisting of a combination of upper- (bent over row, shoulder press), lower- (squat, split squat) and full-body (power clean and press, step and press, pulldown to squat, high pull) exercises (see electronic supplementary material file 1 for images and descriptions of exercises). Participants then completed four sets of high-intensity exercise, with each set consisting of four exercise repetitions. Each set consisted of a combination of four of the exercises previously described with one exercise performed per repetition. In week 1, repetition duration was 45-s with each repetition followed by a 15-s transition period allowing participants to move to the next exercise. Each set was followed by 3 min of passive rest. Repetition duration increased by 10 s at the end of every third week, with duration being 75-s by week 10. Transition period and rest duration remained constant over the course of the intervention with total exercise duration increasing from 12 to 20 min. Each session concluded with a cool down consisting of low-intensity, full-body movements (~ 4 min).

During the intervention exercise order was randomised within- and between-sessions, with participants completing each exercise approximately the same number of times across the 12 weeks. This approach meant that participants could perform the same exercises in multiple consecutive sets, but not in consecutive repetitions, with all exercises not necessarily performed in each session. The aim of each exercise session was to deliver a full-body workout for all participants. As the nature of the Speedflex machine does not permit the setting of a fixed external resistance, participants were asked to work at ‘high-intensity’ during exercise bouts, defined as peak heart rate (HR_peak_) > 90% of maximal heart rate (%HR_max_). Subjects were provided with strong verbal encouragement throughout each HIT session and were instructed to reach target heart rate by increasing the speed of movement during exercises.

Heart rate was monitored continuously during exercise at 5 s intervals using the Polar Team2 system (Polar Electro, Kempele, Finland) with maximal heart rate estimated using the formula: maximal heart rate (HR_max_) = 207 − 0.7 × age [24]. Where a participant exceeded this predicted value during training, HR_max_ was amended to the higher observed value [25]. Using the CR100^®^ scale [26] participants provided differential ratings of perceived exertion (RPE) ~ 10 min after the completion of exercise for upper-body muscle exertion (RPE-U), lower-body muscle exertion (RPE-L) and perceived sense of breathlessness in the chest (RPE-B) [27]. Compared with alternative scales (e.g., Borg 6–20 RPE scale^®^), the CR100^®^ offers a larger and more finely graded numerical range [0–100 AU (arbitrary units)] with several verbal anchors placed along the scale corresponding to whole numbers (0, nothing at all; 12, easy; 22, moderate; 35, somewhat hard; 50, hard; 70, very hard; 100, maximal). The use of the CR100^®^ scale agrees with modern psychophysical theory [26] and allows for a more sensitive assessment of perceived exertion while also having the advantage of providing association to a percentage scale [28]. Participants were habituated with the CR100^®^ scale and the concept of differential RPE at the study outset.

### Outcome measures

At baseline, participants completed three pre-tests over several days (April 2016) with a minimum of 48 h between sessions and at the same time of day to minimise the impact of circadian variation [29]. To reduce systematic bias and stabilise random variability [30] our primary outcome measures (leg extensor muscle power and handgrip strength) were assessed on three separate occasions and our secondary outcome (predicted *V*O_2max_) was assessed twice. Health-related quality of life was evaluated once. Post-intervention (~ 3–7 days following final training session; August 2016) all outcome measures were assessed once. All testing was performed by the same researchers, strictly adhering to the prescribed standardised testing procedures with individual subject data coded to ensure blinding during data analysis. Participants were asked to avoid strenuous physical activity, alcohol and caffeine for 24 h prior to each testing session.

#### Leg extensor muscle power

Leg power was assessed using the Nottingham leg extensor power rig (Medical Engineering Unit, University of Nottingham, Nottingham, UK), a reliable method for assessing lower-body muscular power [21]. Testing was performed in a randomised, counterbalanced order with participants performing all testing sessions in the same order (e.g., always dominant leg first or always non-dominant leg first based on initial randomisation). Participants completed a standardised warm-up consisting of three leg extensions at increasing submaximal intensity (~ 50, ~ 75 and ~ 90% of self-perceived maximal effort). Following this, ten maximal effort leg extensions, each separated by 30 s of passive rest were performed with participants instructed to extend their leg “as hard and as fast as possible”. This process was then repeated on the second leg. The highest value recorded over the ten leg extensions was taken as peak power output (W). Typical error (CV) was small on both the dominant (7.4%) and non-dominant legs (5.6%) for males and females combined after baseline test three [31].

#### Handgrip strength

Handgrip strength was assessed using a digital strain-gauge dynamometer (TKK 5401; Grip-D, Takei Scientific Instruments Co., Ltd., Tokyo, Japan). Testing was performed on both hands with testing order determined as previously described. Participants were instructed to maintain the standard bipedal position, with the arm in complete extension and feet positioned hip width apart. The dynamometer was adjusted appropriate to the individual’s hand size with scores recorded to the nearest 0.1 kg. Following a submaximal practice attempt, participants performed three maximal efforts, with 30 s rest following each attempt. The highest recorded value across the three attempts was used for analysis. Typical error was small on the dominant hand (7.1%) and small on the non-dominant hand (4.6%) after baseline test three.

#### Cardiorespiratory fitness

Cardiorespiratory fitness (predicted *V*O_2max_) was assessed using the Chester Step Test [32], a submaximal, multi-stage step test which has previously been shown to be a reliable tool for the estimation of aerobic capacity [33]. The test consists of five stages, each of 2-min duration with a maximum test time of 10 min. Stepping cadence began at 15 steps/min^−1^ (60 bpm) and increased by 5 steps/min^−1^ at the end of every 2-min stage with step frequency controlled by an electronic metronome (Apple iPad, Apple, California, USA). The test was terminated if the participant reached > 80% of predicted HR_max_ before the completion of stage five. Heart rate (Polar RS400, Polar Electro, Kempele, Finland) was recorded throughout each 2-min stage with maximal heart rate calculated via the equation of Gellish et al. [24]. Following completion of the test, *V*O_2max_ was estimated using the Chester step test calculator (Assist Creative Resources, Wrexham, UK). After the second baseline test in this investigation [33], typical error was small (6.2%).

#### Health-related quality of life

Health-related quality of life (HRQoL) was evaluated using the Short Form-36 health questionnaire (SF-36v2, Optum, Eden Prairie, Minnesota, USA) which contains 36 items used to measure 8 domains of HRQoL [34]. The domains of physical functioning, role limitations due to physical health (role-physical), bodily pain and general health relate to the physical component of HRQoL. Domains of vitality, social functioning, role limitations due to emotional health (role-emotional) and mental health relate to the mental component. The SF-36v2 utilises norm-based scoring (NBS) whereby participant responses to each health domain scale are transformed to a *T* score with a mean value of 50 and a standard deviation of 10. Higher scores indicate better HRQoL.

### Statistical analysis

Prior to all analyses plots of the residuals versus the predicted values revealed no evidence of non-uniformity of error. For training data, the proportion of HIT repetitions that met the pre-specified heart rate criteria for high-intensity was determined, with the median and interquartile range (IQR) for these proportions calculated subsequently. Linear mixed modelling, to allow for fixed (RPE) and random effects (within-participant) was used (SPSS v.23, Armonk, NY, USA: IBM Corp) to examine the difference between differential RPE scores and to determine within-subject variability [expressed as a standard deviation (SD) in RPE-U, RPE-L and RPE-B] with the SD doubled to interpret its magnitude [35].

All outcome measures were log transformed to reduce bias arising from non-uniformity of error and subsequently back transformed to obtain the percent difference between baseline and post-intervention, with uncertainty of the estimates expressed as 90% confidence intervals (CI). An analysis of covariance (ANCOVA) model was used (SPSS v.23, Armonk, NY, USA: IBM Corp) to compare change scores between the two groups [36] with age, sex and baseline value of the outcome measure included as covariates to control for any imbalances between the groups at baseline even after minimisation [37]. Adjusted mean intervention effects were evaluated for their practical/clinical significance by pre-specifying thresholds for small, moderate and large effects [38]. As robust clinical anchors for our outcome measures remain to be determined in this population, magnitude of effects were defined as standardised mean differences of 0.2, 0.6 and 1.2 between-subject standard deviations (SD) for small, moderate and large effects, respectively [39]. The SD of the pooled baseline values was used for this purpose, as the post-intervention SD can be inflated by individual differences in response to the intervention. Using the mean intervention effect for each outcome, together with its uncertainty (i.e., the confidence interval), the probability of the true effect being trivial, beneficial or harmful was calculated, then interpreted using the following scale: < 0.5%, most unlikely or almost certainly not; 0.5–5%, very unlikely; 5–25%, unlikely or probably not; 25–75%, possibly; 75–95%, likely; 95-99.5%, very likely; >99.5%, most likely [39]. Effects were evaluated clinically given that interventions can be potentially harmful as well as beneficial to individuals. The default probabilities for declaring an effect clinically beneficial are < 0.5% (most unlikely) for harmful and > 25% (possibly) for benefit; a clinically unclear effect is, therefore, possibly beneficial (> 25%) with an unacceptable risk of harm (> 0.5%) [39]. Data are presented as mean ± standard deviation (SD) or, for adjusted mean change, 90% confidence interval (CI).

## Results

### Exercise training attendance and intensity

All 18 participants completed the HIT intervention with an overall attendance of 99% (429 out of a possible 432 sessions) across the 12-week period. There were 42 individual sessions rearranged (~ 10%) throughout the intervention period to offset participant unavailability and maximise attendance. Sixteen participants completed all 24 HIT sessions, one participant completed 23 sessions and one participant completed 22. The reasons for missed sessions were: (1) injury unrelated to the study (two sessions missed) and (2) family commitments (one session missed). It was not possible to rearrange these three sessions because of a lack of time during the intervention period. Exercise intensity data are presented in Table 2. The proportion of repetitions meeting the high-intensity criterion [peak heart rate (HR_peak_) ≥ 90% HR_max_] was 62% (IQR 24–81%). There were most likely small differences between RPE-U and RPE-L [6 Arbitrary units (AU); ± 90% confidence limits 1 AU] and RPE-L and RPE-B (6 AU ± 1 AU). The difference between RPE-U and RPE-B was most likely trivial (0 AU; ± 1 AU). The magnitude of the within-subject variability for all RPE measures was moderate. No adverse events were reported during any of the exercise testing or training sessions.

**Table 2:** Training intervention descriptives

	Heart rate (% of maximum)		Rating of perceived exertion (AU)		
	HR_mean_	HR_peak_	RPE-U	RPE-L	RPE-B
Training block 1	82 ± 6	90 ± 7	43 ± 19	36 ± 15	43 ± 19
Training block 2	83 ± 6	90 ± 6	44 ± 18	39 ± 16	46 ± 22
Training block 3	82 ± 6	89 ± 6	43 ± 21	36 ± 15	41 ± 20
Training block 4	81 ± 7	88 ± 7	41 ± 21	34 ± 17	38 ± 20
Overall intervention	82 ± 6	89 ± 6	42 ± 19	36 ± 16	42 ± 20

Data presented as mean ± SD

Training block 1, weeks 1–3; Training block 2, weeks 4–6; Training block 3, weeks 7–9; Training block 4, week 10–12

*HR*_*mean*_ mean heart rate (% of maximum), *HR*_*peak*_ peak heart rate (% of maximum), *AU* arbitrary units, *RPE-U* rating of perceived upper-body muscle exertion, *RPE-L* rating of perceived lower-body muscle exertion, *RPE-B* rating of perceived breathlessness

### Outcome measures

Baseline, adjusted mean change values and between-group comparisons (HIT vs CON) are presented in Table 3. Compared to CON, HIT showed possibly small beneficial effects for dominant leg power, non-dominant leg power and non-dominant handgrip strength. For dominant handgrip strength, the effect was likely trivial. There was a likely small beneficial effect for predicted *V*O_2max_.

**Table 3:** Baseline, adjusted mean change and between-group comparison for leg extensor muscle power, grip strength and cardiorespiratory fitness

	Intervention (HIT; *n* = 18)		Control (CON; *n* = 18)		Group comparison (HIT–CON)	
	Baseline value (mean ± SD)	Adjusted mean change^a^ (% mean; 90% CI)	Baseline value (mean ± SD)	Adjusted mean change^a^ (% mean; 90% CI)	Between group difference (% mean; 90% CI)	Qualitative inference
Leg extensor muscle power						
Dominant leg (W)	159.2 ± 64.8	10.6; 3.8 to 17.8	161.8 ± 63.2	0.1; − 4.3 to 4.7	10.5; 2.4 to 19.4	Possibly small beneficial
Non-dominant leg (W)	166.7 ± 57.9	10.4; 5.1 to 16.1	175.1 ± 68.3	0.9; − 2.3 to 4.3	9.4; 3.3 to 16.0	Possibly small beneficial
Handgrip strength						
Dominant hand (kg)	36.2 ± 10.9	4.2; 0.5 to 8.1	33.9 ± 11.0	− 1.5; − 5.1 to 2.1	5.9; 0.5 to 11.5	Likely trivial
Non-dominant hand (kg)	33.6 ± 10.8	5.0; 1.5 to 8.7	31.2 ± 9.4	− 1.1; − 4.4 to 2.3	6.3; 1.2 to 11.5	Possibly small beneficial
Cardiorespiratory fitness						
Predicted *V*O_2max_ (mL · kg^−1^ · min^−1^)	33.8 ± 8.3	11.5; 5.8 to 17.5	33.9 ± 5.4	2.8; − 0.8 to 6.7	8.4; 1.8 to 15.4	Likely small beneficial

*HIT* high-intensity interval training, *CON* no-exercise control group, *CI* confidence interval

^a^Analysis adjusted for age, sex and baseline value

For health-related quality of life (Table 4), there were possibly small beneficial effects for role-physical, general health, vitality and mental health and a likely small beneficial effect for bodily pain in the HIT group compared with CON. There was a possibly moderate beneficial effect for role-emotional. Between-group differences in physical functioning and social functioning were likely and possibly trivial, respectively.

**Table 4:** Baseline, adjusted mean change and between-group comparison for health-related quality of life

	Intervention (HIT; *n* = 18)		Control (CON; *n* = 18)		Group comparison (HIT–CON)	
	Baseline (mean ± SD)	Adjusted mean change^a^; 90% CI	Baseline (mean ± SD)	Adjusted mean change^a^; 90% CI	Between group difference; 90% CI	Qualitative inference
Physical functioning	53.2 ± 3.6	0.4; − 0.9 to 1.7	53.5 ± 4.0	0.6; − 0.5 to 1.7	− 0.2; − 1.8 to 1.5	Likely trivial
Role-physical	50.0 ± 5.8	3.7; 1.4 to 6.1	51.2 ± 4.9	0.8; − 1.6 to 3.1	3.0; − 0.4 to 6.3	Possibly small beneficial
Bodily pain	52.9 ± 8.3	3.1; − 0.5 to 6.8	54.3 ± 5.9	− 1.1; − 3.4 to 1.1	4.3; 0.0 to 8.5	Likely small beneficial
General health	53.0 ± 7.6	2.0; − 0.4 to 4.5	56.4 ± 6.6	− 1.3; 1.0 to 2.3	3.3; 0.0 to 6.6	Possibly small beneficial
Vitality	55.1 ± 7.4	2.7; − 0.2 to 5.5	54.9 ± 4.9	− 0.5; − 3.4 to 2.3	3.2; − 0.8 to 7.2	Possibly small beneficial
Social functioning	53.7 ± 6.6	− 0.4; − 2.8 to 2.1	55.4 ± 5.2	0.3; − 1.9 to 2.5	− 0.7; − 3.9 to 2.5	Possibly trivial
Role-emotional	55.8 ± 1.1	1.1; − 1.5 to 3.8	54.0 ± 3.6	− 3.8; − 6.5 to 1.2	4.9; 1.1 to 8.8	Possibly moderate beneficial
Mental health	53.0 ± 6.2	2.9; 0.7 to 5.0	53.6 ± 7.1	− 0.1; − 2.3 to 2.1	2.9; − 0.1 to 6.0	Possibly small beneficial

Data are presented as norm-based scores (NBS)

*HIT* high-intensity interval training, *CON* no-exercise control group, *CI* confidence interval

^a^Analysis adjusted for age, sex and baseline value

## Discussion

The present investigation has evaluated the effects of 12-weeks of combined upper- and lower-body HIT on muscular and cardiorespiratory fitness in older adults observing possibly beneficial improvements in leg power and handgrip strength as well as a likely beneficial improvement in cardiorespiratory fitness. These data provide support for HIT as a multicomponent training strategy in older adults.

Notwithstanding any potential training induced physiological and performance improvements, exercise training interventions must be acceptable and safe for participants. Despite there being no adverse events recorded during the training programme, the limited sample size and training programme duration mean that considerably more data are needed to fully quantify the risks associated with this training approach in older adults. Evaluation of attendance and adherence (i.e., meeting the prescribed exercise intensity [40]) can provide a useful quantification of the feasibility of an intervention. The attendance for the 18 participants across the 12-week intervention reported in this study is considerably higher than the 58–77% range for older adults undertaking exercise programmes reported in the systematic review of Picorelli and colleagues [41]. It is acknowledged however, that the structure of this training intervention—with extensive availability of the instructor and the exercise facility—meant a flexible approach to exercise session timing was possible to promote and achieve maximal possible attendance for all participants. Therefore, the present data represent a ‘best case’ scenario which may be considerably different from a ‘real-world’ scenario. Importantly, however, these data do provide support for the feasibility of instructor-led group-based HIT as a method for engaging older adults if a flexible approach to session delivery is possible.

As well as attendance, assessing if participants have performed the exercise training as intended is essential when evaluating interventions [40]. Using our pre-specified target threshold for high-intensity, 62% of repetitions met the criteria. Previous evaluation of intervention fidelity has suggested that high-intensity criterion attainment in 58% of HIT repetitions represented ‘moderate’ intervention fidelity [40], while Weston et al. [27] reported ‘low’ fidelity with ~ 23% compliance to HIT criteria. Based on these previous interpretations, it seems reasonable to categorise intervention fidelity as ‘moderate’ in this study. Despite this however, between-study comparisons of intervention fidelity remain limited by a lack of available data. Wider translation of this approach to evaluating the fidelity of exercise training interventions may be enhanced through the use of clear thresholds and accompanying qualitative descriptors. As such, it is recommended that as per Taylor et al. [40] authors report the median along with the interquartile range of sessions/bouts meeting pre-specified criteria and qualify this using our proposed fidelity thresholds and corresponding qualitative descriptors (median number of sessions/bouts meeting pre-specified criteria: < 50%, low; 50–70%, moderate; > 70%, high). The thresholds suggested here represent the authors’ personal experiences of exercise training prescription and evaluation. Further investigation is needed to appraise these qualitative descriptors and their corresponding thresholds.

Mean RPE scores in this investigation fell in the range of ‘somewhat hard’ to ‘hard’, lower than previous studies prescribing HIT intensity as ‘hard’ to ‘very hard’ [27, 42] but similar to those reported following HIT in overweight young adults [43]. Between-study differences are likely multifactorial in nature with heterogeneous study populations as well as differences in exercise programming factors contributing to these observed differences [44]. Despite lower perceived exertion responses than typically prescribed during HIT, the present investigation has reported a clear beneficial effect on predicted *V*O_2max_—a finding with clear practical implications as exercise that is perceived to be less intense may be more palatable to potential exercisers [43].

Possibly small beneficial improvements in leg power on both the dominant and non-dominant leg were observed in this investigation. This finding has clear practical implications as improvements in leg power have been shown to make an important contribution to clinically meaningful improvements in gait speed and are associated with improved functional performance [2–4]. Previous investigations have shown potential for HIT to increase muscular power [15, 41]; however, these studies have typically employed training protocols classified as sprint-interval training (SIT) or performed at ‘all out’ intensity which may not be suitable or appealing to all. The present findings demonstrate that HIT does not need to be performed ‘all out’, with submaximal HIT capable of inducing improvements in muscular power. This finding may be of particular relevance in this population, where adherence to physical activity guidelines is poor [9], as previous work has suggested that exercise that is too strenuous is likely to be a deterrent to participation [45]. The observed improvements in muscular power are of a similar magnitude (~ 10%) to those previously reported in young [46, 47] and older adults [16]. However, these studies assessed peak power using either the Wingate, or a maximal incremental test and as Sculthorpe et al. [15] have noted, the mechanical and metabolic differences between single expressions of power (e.g., during a leg extension) versus high-intensity cycling mean that between-study comparisons are limited. Our study is the first to demonstrate that submaximal HIT increases explosive lower-body muscle power in older adults. Further work is needed to understand the mechanistic basis of this adaptation.

Previous investigation has shown handgrip strength to be a strong predictor of mortality [48], while a reduction in grip strength is also related to difficulty in performing activities of daily living [1]. The present data indicate a possibly beneficial improvement in handgrip strength on the non-dominant hand of a similar magnitude to Pereira et al. [49] who reported improvements of 5% (dominant hand) and 6.9% (non-dominant hand) after 12-weeks of high-speed power training. Interestingly, both the current study and Pereira et al. [49] have shown greater improvements in handgrip strength on the non-dominant hand. This may be related to lower levels of baseline strength on the non-dominant hand as improvements in strength are related to initial strength level with greater increases reported in participants with lower baseline levels [50]. Our findings demonstrate that HIT could be an effective approach to increase handgrip strength when exercises are prescribed to target upper-body muscle groups. This supports the idea that creative approaches to exercise prescription are needed to maximise the potential for functionally relevant training induced adaptations.

The robust relationship between greater cardiorespiratory fitness and reduced mortality and morbidity means that the likely small beneficial improvement in predicted *V*O_2max_ reported in this investigation is an important and clinically relevant finding [5]. The observed improvement in *V*O_2max_ falls within the range of previous studies investigating HIT in older adults, employing the archetypal 4 × 4 min protocol of 6–15% [13, 14, 16]. It should be noted, however, that the study reporting the largest improvement (15%; [13]) involved a 10-week training programme with three training sessions per week equating to 25% more training sessions performed than in the current investigation. As lack of time remains a commonly cited barrier to exercise participation [10], training programmes which require a reduced time commitment, by prescribing a lower number of weekly sessions, may be more appealing to potential exercisers. Caution is also warranted when making between-study comparisons as improvements in *V*O_2max_ are affected by baseline fitness, with greater benefit observed for less fit participants [18]. Although speculative, it is possible that both central and peripheral adaptations contribute to increased cardiorespiratory fitness following HIT as reported in this investigation [51].

The present data suggest that HIT has potential to improve perceptions of health-related quality of life in older adults—an important aim for health interventions involving this population group [52]. Although previous work has suggested that HIT may have a beneficial effect on health-related quality of life in healthy [53] and clinical populations [54], comparison of our findings with previous investigations is challenging because of differences in methods of assessment and heterogeneous study populations. For example, the participants in the present investigation exhibited baseline values considerably higher than the mean across seven of the eight health domains. It is likely that participants with higher perception of health-related quality of life at baseline will need to demonstrate improvements of a greater magnitude, compared with those reporting lower values at baseline, to have a meaningful effect. However, there remains no consensus on what represents the minimal clinically relevant change in health-related quality of life for healthy older adults and further work, involving longer duration follow-up should attempt to elucidate this. Caution is also warranted when interpreting our findings as short-term improvements in health-related quality of life may not translate into long-term changes.

Although this study has demonstrated that 12-weeks of HIT can elicit improvements in muscular power and strength as well as cardiorespiratory fitness, it is not without limitation. Firstly, this is a pragmatic, exploratory trial involving only a small number of participants. It is acknowledged that studies with low statistical power involving small sample sizes likely overestimate or exaggerate the magnitude of an effect [55]. A further definitive trial involving a greater number of participants is needed to accurately quantify training induced changes using this exercise mode. Moreover, a larger sample size would also allow for exploration of potential moderator variables (e.g., sex) which may influence the observed training response. Secondly, the outcome measures used in this investigation provide an understanding of the effects of HIT on isolated components of physical fitness (e.g., lower-body muscle power, upper-body muscle strength, cardiorespiratory fitness), yet performance on functional fitness tests (e.g., 30-s chair stand test, timed up and go) may be more relevant for assessing intervention effects as these tests provide composite measures of physical performance [56]. In addition, submaximal estimation of *V*O_2max_ is acknowledged as being an alternative to the gold standard approach for assessing cardiorespiratory fitness. However, the Chester Step test does quantify cardiorespiratory fitness in terms of *V*O_2max_, facilitating comparison with previous investigations. Although laboratory-based determination of *V*O_2max_ via incremental exercise testing is the benchmark for assessing cardiorespiratory fitness, the pragmatic nature and logistical constrains of this investigation meant this was not possible. A future definitive trial should include measures of functional fitness and laboratory-based determination of *V*O_2max_, while an increased follow-up period may help to understand the longer-term effects of this training approach (i.e., the maintenance of training induced changes in fitness over time) which were not evaluated in the present investigation. Finally, the results of the present investigation are representative of a healthy and relatively young older adult population, and therefore, should not be extrapolated to represent all older adults, including those with multimorbidity as well as individuals engaged in drug therapy. Consequently, further work is needed to evaluate the potential effectiveness of this training approach with these population groups.

## Conclusions

Our study has demonstrated that 12-weeks of combined upper- and lower-body HIT is an effective method for inducing clinically meaningful improvements in aspects of muscular and cardiorespiratory fitness in adults aged over 50 years. In particular, the observed improvements in lower-body muscle power are especially relevant because of the role this plays in maintaining functional performance in this population. Moreover, the present data provide evidence that HIT does not need to be performed at an ‘all out’ intensity to induce fitness improvements in this population. More broadly, this study provides further indication that HIT is a feasible and effective approach to exercise training in older adults with high intervention fidelity reported. These findings highlight potential for innovative approaches to training delivery and should encourage researchers to move beyond exercise modes traditionally associated with HIT.

## Electronic supplementary material

Below is the link to the electronic supplementary material.

Supplementary material 1 (PDF 6223 KB)
