# Supplementary material for: The effect of 12 weeks of combined upper- and lower-body high-intensity interval training on muscular and cardiorespiratory fitness in older adults
Source: Aging Clin Exp Res. 2018 Jul 26;31(5):661–71. doi: 10.1007/s40520-018-1015-9 (PMC6491660; doi:10.1007/s40520-018-1015-9)
Supplement: Supplementary file 1 — Supplementary material 1 (PDF 6223 KB) [file 40520_2018_1015_MOESM1_ESM.pdf]

Article title: The effect of twelve weeks of combined upper- and lower-body high intensity interval training on muscular and cardiorespiratory fitness in older adults

Journal: Aging Clinical and Experimental Research

Authors: Christopher Hurst[1,2], Kathryn L Weston[3] & Matthew Weston[2]

Affiliations: [1]School of Applied Social Sciences, Durham University, Durham, United Kingdom.
[2]Department of Psychology, Sport & Exercise, School of Social Sciences, Humanities and Law, Teesside University, Middlesbrough, United Kingdom.
[3]School of Health and Social Care, Teesside University, Middlesbrough, United Kingdom.

Corresponding Author: Christopher Hurst
School of Applied Social Sciences
Durham University
42 Old Elvet
Durham
DH1 3HN
UNITED KINGDOM

Email: christopher.d.hurst@dur.ac.uk
Telephone: +44 (0)191 3341516
Fax: +44 (0)191 3346987

Name: **Supplementary file 1. Diagrams and descriptions of exercises performed.**

Squat

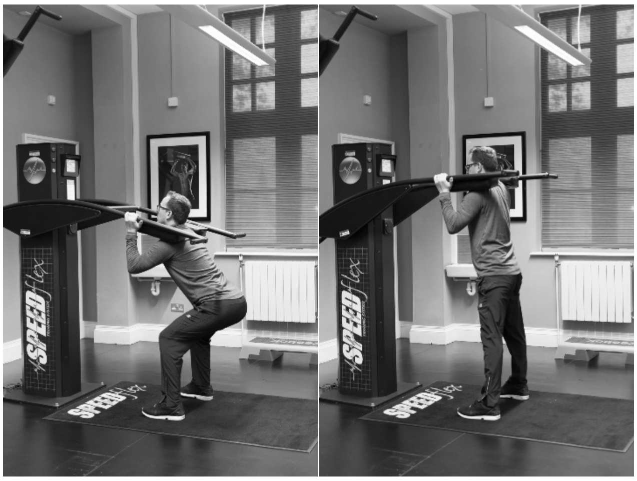

Exercise description:

Participant begins by standing with feet hip width apart or slightly wider with toes pointing forwards with the pads of the machine resting on shoulders. Participant should keep the head and chest up looking straight forward. Participant should keep a flat or naturally slightly arched back. Allowing knees to bend slowly for the downwards phase of the movement, the participant should make sure to keep heels on the floor throughout. Once at the bottom of the movement, participant should extend the hips and knees together at the same speed. Make sure the shoulder pads remain in contact with shoulders at all times.

Split-squat

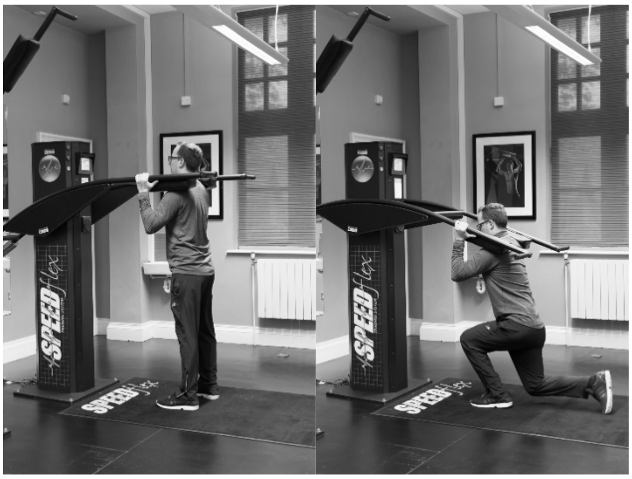

Exercise description:

Participant begins by standing with feet hip width apart or slightly wider with toes pointing forwards with the pads of the machine resting on shoulders. Keep the head and chest up looking straight forward. Participant should keep a flat or naturally slightly arched back. Squat down by flexing knee and hip of front leg. Allow heel of rear foot to rise up while knee of rear leg bends slightly until it almost makes contact with floor. Return to original standing position by extending hip and knee of forward leg. Repeat. Continue with opposite leg. Make sure the shoulder pads remain in contact with shoulders at all times.

Bent over row

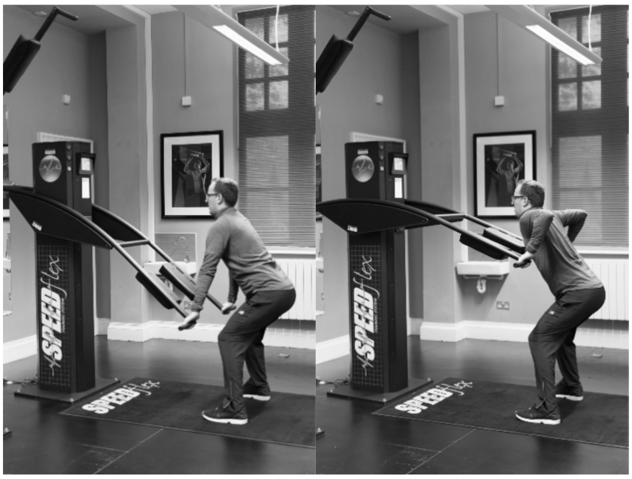

Exercise description:

Participant holds the bar with feet shoulder/hip width apart. Pushing the hips back and letting the knees bend slightly with the bar at about knee height. The back should be flat. Participant should hold this position keeping the lower body still. Participant should drive the hands up towards the chest (under the arm pit) keeping the back flat. The arms should then be fully extended to return to the starting position.

Shoulder press

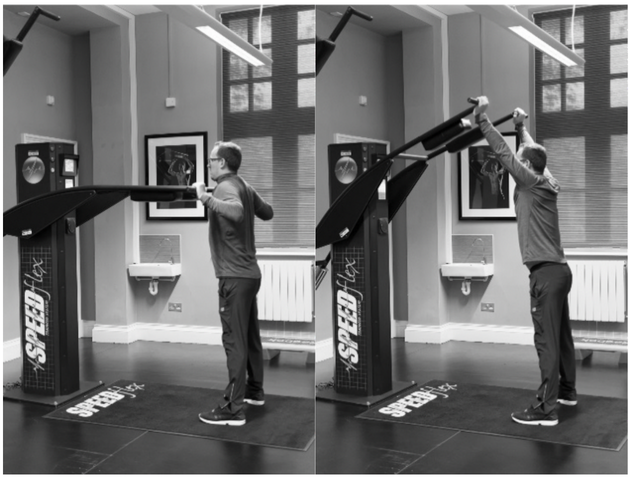

Exercise description:

Participant begins by standing with feet hip width apart or slightly wider with toes pointing forwards. From shoulder height, participant presses the bar above the head, fully extending the arms. From the top position, participant pulls the bar back to shoulder height.

Power clean and press

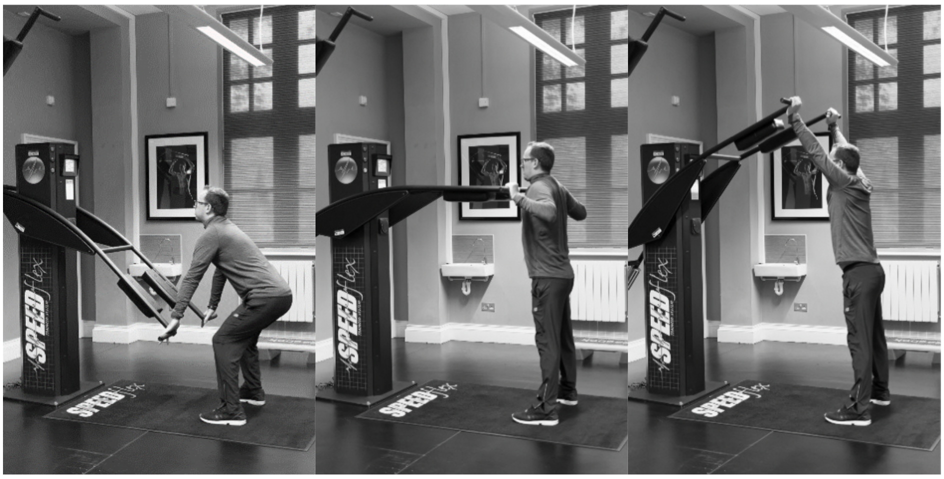

Exercise description:

Participant starts holding the handles of the machine with feet shoulder width apart. Movement begins by bending at the knees, keeping back flat and arms fully extended. Keeping arms extended, participant lifts the bar by standing up extending hips and knees. As the bar approaches chest height,

participant bends knees and brings the bar to shoulder height. From shoulder height, participant presses the bar above the head, fully extending the arms. From the top position, participant pulls the bar back to shoulder height and reverses the movement.

Step and press

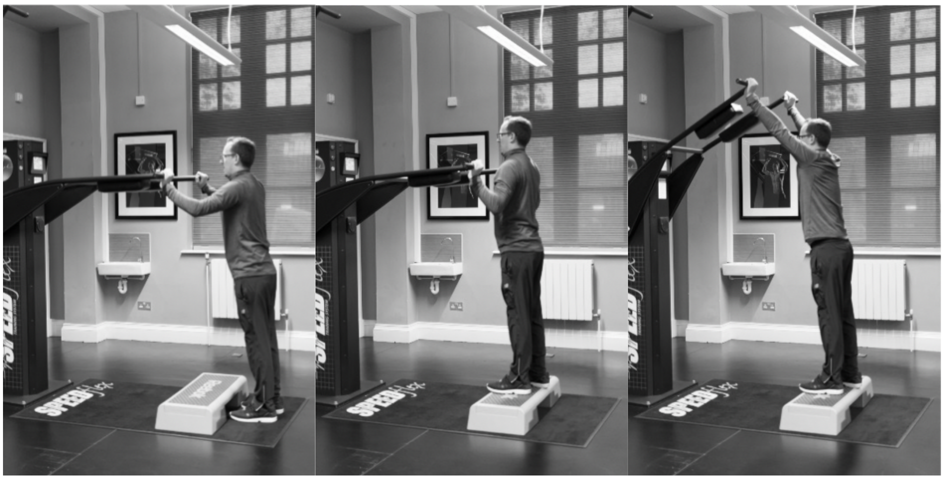

Exercise description:

Participant starts with both feet on the floor and bar at shoulder height. Participant step both feet up (one at a time) and onto the box. Participant should press the bar above the head (i.e. fully extend arms) at the same time as stepping until the arms are fully extended. Once at the top of the movement, participant should step back down and pull the bar back to the starting position.

Pulldown to squat

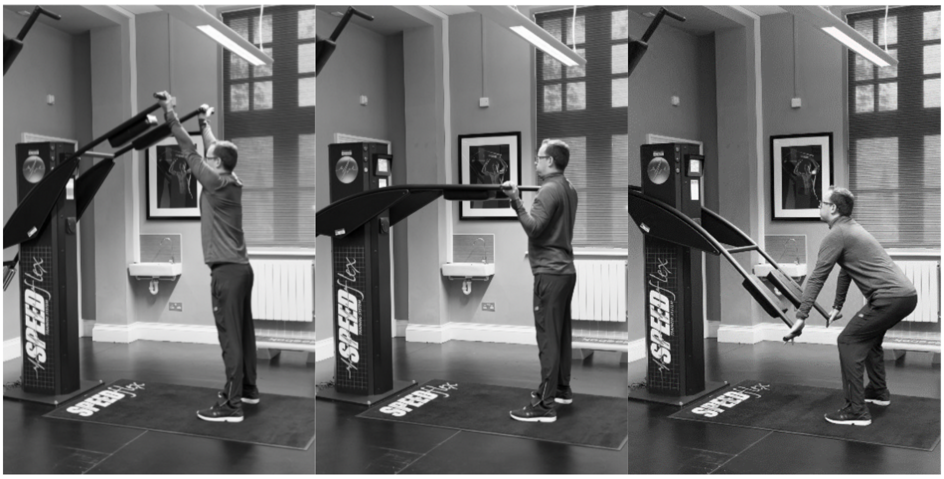

Exercise description:

Movement starts with participant’s arms fully extended holding the handles with a supinated grip and feet hip width apart. Looking straight forward the participant should pull the bar down to shoulder height (the pulldown phase of the exercise). Keeping hands in line with shoulders and back in a neutral position with a slight arch lower into a squat phase. Participant should allow the knees to bend and keep heels on the floor at all times. Once at the bottom of the movement, participant should extend the hips and knees together at the same rate making sure hands are still in line with shoulders. Once the participant is standing tall press the bar above the head.

High pull

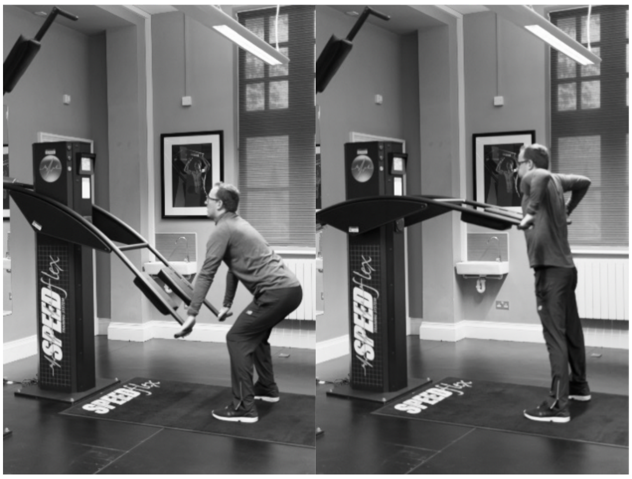

Exercise description:

Participant holds the bar with feet shoulder width apart. Pushing the hips back and letting the knees bend slightly as the bar lowers to about knee height. The back should be flat. Participant should explosively thrust the hips forward simultaneously pulling the bar to lower chest height with the elbows high. At the peak bar position heels can either remain on the floor or for more range participants can come up onto the toes. Participant should push the bar back down reversing the process in a smooth, rapid fashion as heels return to the floor. Hips push back, and the back stays flat.
